# Pursuit dynamics among Australian marsupials and dogs: comparative locomotor performance and escape probability

**DOI:** 10.1242/jeb.252204

**Published:** 2026-10-05

**Authors:** Joshua L. Gaschk, Kaylah Del Simone, Ami Fadhillah Amir Abdul Nasir, Mikaela J. Bratovic, Robbie S. Wilson, Christofer J. Clemente

**Affiliations:** ^1^ School of BioSciences, University of Melbourne, Parkville, VIC 3010, Australia; ^2^ School of Science, Technology and Engineering, University of the Sunshine Coast, Sippy Downs, QLD 4556, Australia; ^3^ School of Biological Sciences, University of Queensland, St Lucia, QLD 4067, Australia; ^4^ School of Biomedical Sciences, University of Queensland, St Lucia, QLD 4067, Australia

**Keywords:** Agility, Canids, Dasyurids, Escape probability, Locomotion, Peramelids

## Abstract

Predation can strongly influence population outcomes, and locomotor performance may be especially important during pursuit–evasion interactions. We hypothesised that differences in performance-based escape capacity could contribute to the abundance and distribution of prey populations. We compared the locomotor performance of northern brown bandicoots (*Isoodon macrourus*), which persist across mainland Australia, and northern quolls (*Dasyurus hallucatus*), which have declined on the mainland, with that of domestic dogs (*Canis familiaris*; multiple breeds, 3.4–44 kg) as an approximation of canid performance. Velocity, acceleration and changes in heading were extracted from top-down video footage and used to estimate relative escape performance. Dogs achieved the highest mean and maximum speeds, whereas quolls showed the greatest turning capacity. Bandicoots were the slowest, least agile and most predictable of the three groups. The ability of quolls to outmanoeuvre a pursuing predator may help explain their association with structurally complex habitats, where agility should provide a greater advantage. By contrast, the persistence and abundance of northern brown bandicoots are likely supported by other ecological traits because their locomotor capacity for avoiding pursuit predators appears limited. These findings show that locomotor performance alone cannot explain prey population outcomes, but may interact with habitat complexity to favour different escape strategies.

## Teaser

**Highlighted Article:** Comparison of locomotor performance between Australian fauna and an invasive predator suggests that escape probability alone cannot explain prey distribution and abundance; rather, locomotor strategy may interact with habitat to favour species survivability in complex environments.

## INTRODUCTION

Predation greatly influences the structure of an ecosystem through its direct effect on a community, driving natural selection. Predation events have been categorised as ambush, ballistic or pursuit interactions (Moore and Biewener, 2015). Ambush attacks are described as one motion from a concealed predator initiated by one key stimulus from the often-unaware prey. Ballistic interception requires the predator to predict the path of the prey and commit to a single trajectory to intercept, with discrete success or failure. In pursuit predation, the interaction between predator and prey is extended, involving multiple exchanges of stimuli (Clemente and Wilson, 2015b; Moore and Biewener, 2015; Wilson et al., 2018).

The outcome of these predation types depends on the relative performance between the predator and prey. For both ambush and ballistic predators, initial bursts of acceleration and speed are an important component for success, and so maximum speed has been recorded for numerous species (Garland, 1983). Pursuit predation events are characterised by high-speed turning, interspersed with moments of acceleration and deceleration (Howland, 1974; Garland, 1983; Wilson et al., 2013; Clemente and Wilson, 2015a). The prey determines many aspects within a pursuit that relate to turning (e.g. timing of turns, speed through the turns); to be successful, the predator must respond to their prey's stimuli, maximise opportunity but minimise wasted energy (Lima and Dill, 1990; Clemente and Wilson, 2015b; Wilson et al., 2015, 2018). Thus, pursuit predation outcomes will depend on both the relative speed and turning ability of participants (Clemente and Wilson, 2015b; Wilson et al., 2015).

Predicting the outcome of pursuits is complicated further by the biomechanical trade-offs between speed and turning ability. Several studies have illustrated these trade-offs in a variety of terrestrial species including quolls (*Dasyurus hallucatus)*, antechinus (*Antechinus mysticus*), cheetahs (*Acinonyx jubatus*), dogs (*Canis familiaris*) and horses (*Equus caballus*) (Tan and Wilson, 2011; Wilson et al., 2013; Wynn et al., 2015; Wheatley et al., 2018; Haagensen et al., 2022). Four key challenges of turning at high speed are limb force limitations, reorientation of the body, leaning during a turn and coefficient of friction (Haagensen et al., 2022). Turning requires force production in both tangential and centripetal axes and is limited by the amount of force that can be produced by the limbs; at maximum speed, an increase in centripetal force results in a decrease of tangential force (Greene, 1985; Weyand et al., 2000). Therefore, reorienting the body to the new heading angle becomes important as limbs function better in the parasagittal axis, which is parallel to tangential forces (Walter, 2003). Leaning into a turn is an alternative way to achieve this alignment; however, the appropriate level of force leading into the turn is required to avoid lateral toppling (Biewener and Patek, 2018). The level of force that is transferable to the substrate is determined by the coefficient of friction, which is affected by foot/pad morphology and the substrate itself (Russell and Bryant, 2001; Tan and Wilson, 2011; Wynn et al., 2015; Wheatley et al., 2018). To increase turning ability, individuals must overcome these challenges; however, mass and gait of an individual also contribute to success.

Body size is also important in determining turning ability. A mathematical model comparing the probability of capture with different sizes of predator (30 kg, 250 kg) and prey (3–200 kg) showed that the largest predator was able to catch all prey when only straight-line pursuits were simulated; however, when turning pursuits were simulated, the largest predator was unable to catch smaller prey (Wilson et al., 2015). During these same turning pursuits, the smaller predator was able to capture all except the smallest prey (3 kg). A study of turning in domestic dogs revealed that medium-sized dogs (10–25 kg) had a greater capacity for tangential (straight line) and centripetal acceleration (Haagensen et al., 2022). Smaller dogs (<10 kg) were limited by their force production, while large dogs (>25 kg) were limited in their ability to produce centripetal acceleration. Other aspects contribute to effective turning in the wild, including mistakes and the fear of making them (Amir Abdul Nasir et al., 2017; Wheatley et al., 2021), running at suboptimal speeds (Wilson et al., 2013), turning strategies (e.g. timing) (Wilson et al., 2015) and morphological adaptations (e.g. foot or claw morphology); however, much less is known about how the different morphologies and gait affect turning ability (Jindrich and Qiao, 2009).

In Australia, invasive predators have been implicated in the extinction and decline of native species (Doherty, 2015; Woinarski, Burbidge and Harrison, 2015; Doherty et al., 2019; Fleming et al., 2021). This is particularly true for species within the critical weight range (CWR), which proposes a trend in Australia in which many terrestrial species between 35 and 5500 g have gone extinct in comparison to those outside that range (Chisholm and Taylor, 2007). The difference in locomotor performance has been suggested as a potential explanation for the success these invasive predators possess when hunting Australian fauna (Banks and Dickman, 2007; Gaschk and Clemente, 2022). The effect of predation is compounded by land clearing, fire, climate change and habitat degradation, among other contributing factors (Allen, 2011; Griffiths and Brook, 2014; Johnstone, Lill and Reina, 2014; Murphy et al., 2019). These habitat changes likely have an effect on terrestrial locomotion and predator–prey interactions (Winchell, Battles and Moore, 2020).

The complicated nature of a species ecology means that decline of populations has not been consistent across species, with many native fauna remaining relatively unaffected, despite falling within the CWR (Radford et al., 2018; Gaschk and Clemente, 2022). Northern brown bandicoots (*Isoodon macrourus* – from here on bandicoots; 500–3100 g) are strictly terrestrial, and within the CWR. Despite this, they show minimal range extirpation and are of least concern (Lunney et al., 2016). In contrast, northern quolls (*D. hallucatus –* from here on quolls; 300–900 g) are endangered and their distribution has been reduced to fragmented populations across northern Australia (Braithwaite and Begg, 1995; Oakwood, Woinarski and Burnett, 2016).

Dingoes and wild dogs are common predators whose distribution overlaps with that of the bandicoots and quolls but the extent to which they effectively pursue these two prey species is unknown. Both species are present in the diets of dingoes and wild dogs in the tropical and subtropical bioregions (Gaschk and Clemente, 2022). If locomotor performance (speed and manoeuvrability) is an important determinant of escape probability from canid predation pressure, this would increase the number of individuals that survive for multiple seasons compared with a species that does not rely on locomotor performance. We hypothesised that bandicoots would display a greater escape probability in comparison with quolls, reflected in higher top speeds or accelerations. The turning performance of female quolls has been linked to survival in subsequent seasons; however, this has not been quantified and compared directly with that of canids, and no link has been made with bandicoots from the same island (Rew-Duffy et al., 2020). As such, we predicted that quolls would have a greater turning ability in relation to dogs because of their smaller size and suggested importance. If we do not see an effective escape probability for prey species, we can assume some other aspect of their ecology is important for individual and population survival.

## MATERIALS AND METHODS

### Video collation

Videos of bandicoots and quolls were sourced from the Wilson Performance Lab, University of Queensland. Quoll videos were obtained from Wynn et al. (2015); quolls, *Dasyurus hallucatus* Gould 1842, were captured on Groote Eylandt (Northern Territory, Australia) and run on a trackway constructed using two tracks (L: 3.4 m, H: 1.23 m, W: 0.4 m), joined via a removable angle structure, creating a turning lane that was confined to approximately 0.15 m. The corner of the track was adjusted to 135 deg, 90 deg and 45 deg, and the floor was covered in a rubber matting. Bandicoot videos were sourced from Del Simone (2024); bandicoots, *Isoodon macrourus* (Gould 1842), were filmed on Groote Eylandt and at Hidden Vale Wildlife Centre. Bandicoots ran down a trackway constructed of star pickets with shade cloth pulled tight between/around each picket to create the sides (width 0.75 m). This trackway opened into a larger area, which was constructed with the same boundary as the trackway, allowing the bandicoots to determine the direction and angle of their turns. Bandicoots were run on a loamy soil or a clay/grassy substrate. Both quolls and bandicoots were filmed via a mounted camera at the apex of the turn at 240 frames s^−1^ (Casio EXILIM HS EX-ZR200, 512×384). Both bandicoots and quolls were encouraged to run by the presence of a human, taking advantage of their instinct to flee from predation. Dog videos were sourced from The Biomechanics and Biorobotics Lab, University of the Sunshine Coast (3.4–44 kg; for list of breeds, see Haagensen et al., 2022). Dogs, *Canis familiaris* Linnaeus 1758, were run on a grass field around a varying number of cones situated in a turning arc; the number of cones determined the severity of the turn. Dogs were encouraged to run by their trainers – they were either chasing an item (e.g. ball) or following the direction of the trainer. Trained domestic dogs were used to understand maximum speeds and turning performance as an approximation for performance in wild dogs or dingoes. Prompting dogs to chase objects or take orders simulates the hunting of prey and, as such, runs undertaken using this methodology would show speeds and turning ability closely associated with that of wild hunting dogs. More details on methods of animal capture, ethics and permits can be found in the respective papers and a list of dog breeds can be found in Table S2.

### Video analysis

Videos were annotated in 2-dimensions, in MATLAB using DLTdv8 (v.8.0) (Hedrick, 2008). Three points (nose, tail, hind footfall; Fig. 1A) were annotated on the animal throughout the turning sequence, with an additional two points at the start of each sequence for calibration. Calibration points were annotated at a known distance apart. For the agility dogs, this was a measurement on the ground [Dog (D):1.206 m, 0.057 pixels mm^−1^], for the quolls and bandicoots this was the track apex width [Quoll (Q): 0.15 m, 0.311 pixels mm^−1^; Bandicoot (B): 0.75 m, 0.147 pixels mm^−1^]. The outside (opposite side to turning direction) hindfoot was annotated when contacting the ground (footfall). A stride was considered from one hindfoot footfall to the subsequent hindfoot footfall and each performance metric was calculated on individual strides. Nose and tail points were annotated continuously for each frame throughout a turn: on the tip of the nose and the base of the tail. In dogs, because of the wider field of view, the tail and the nose were tagged sequentially from the first footfall until acceleration occurred out of the turn. All individuals were tagged at least one stride before and after the apex of the turn.

**Fig. 1.**
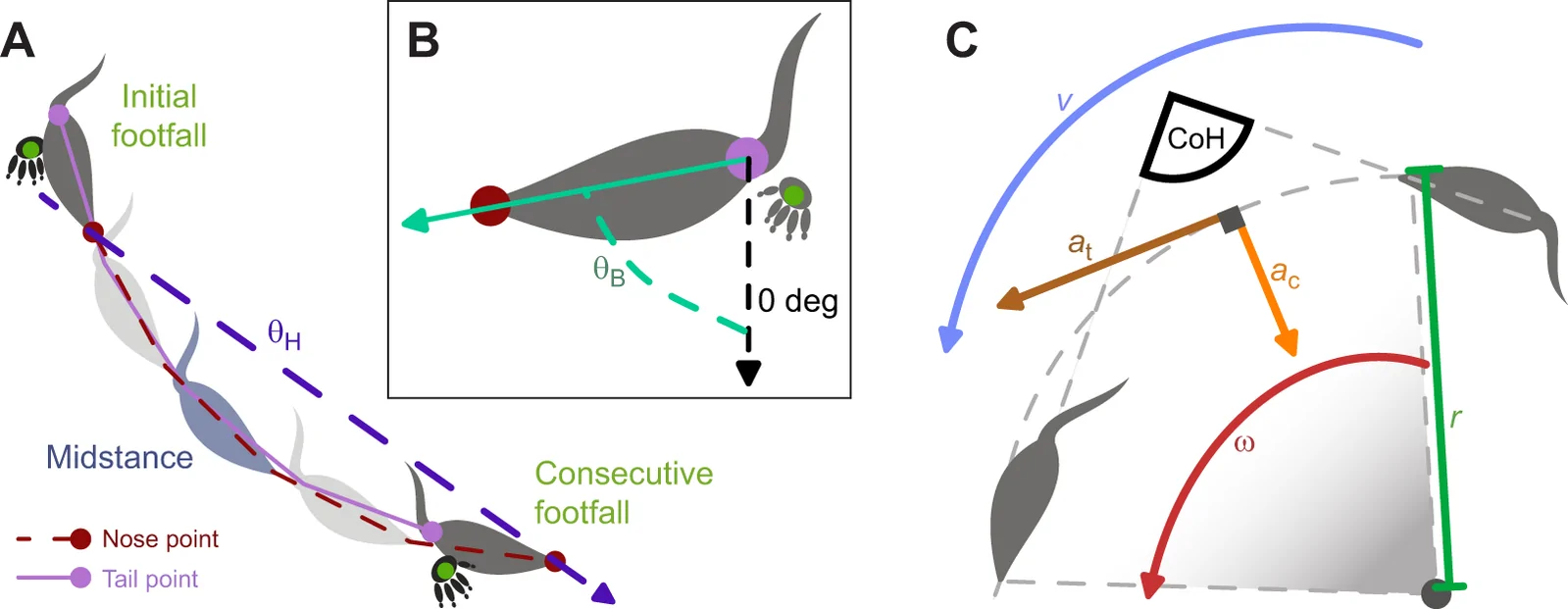
**Visual display of video analysis and calculated metrics.** (A) Annotated points included the nose (maroon), tail (pink) and footfalls (green). The purple line shows the points used to calculate heading angle, θ_H_. (B) Calculation of body angle (θ_B_, teal) relative to 0 deg as the negative *y*-axis. (C) Metrics used for analysis of turning performance: velocity (*v*, blue), angular velocity (ω, red), turn radius (*r*, green), change of heading (CoH, black), tangential acceleration (*a*_t_, brown) and centripetal acceleration (*a*_c_, orange).

### Calculations for turning variables

Stride length (m) was calculated by summing the pixel distance between successive nose points throughout a stride (footfall–footfall) and converting to metres. Stride duration (s) was calculated by totalling the number of video frames of a stride and dividing by the number of frames per second. Frequency (Hz) was calculated as 1/duration. Velocity (*v*, m s^−1^) of a stride was calculated by dividing stride length by the duration of each stride. The between stride velocity (*v*_BS_, m s^−1^) was calculated by finding the difference in velocity between two consecutive strides, which also revealed acceleration and deceleration between strides. Midstance, the halfway point within a stride (Fig. 1A), was found by halving each stride duration (in frames) and adding it to the frame number from the original footfall. Midstance was used to calculate midstance duration (s), midstance length (m) and midstance velocity (m s^−1^). These variables were not formally analysed but were used to create other variables. Tangential acceleration (*a*_t_, m s^−2^), the acceleration tangential to the turn (fore–aft), was calculated by dividing the *v*_BS_ by the midstance duration.

Body angle (θ_B_, deg) was calculated by finding the angle of the line that joins the tail and nose point within a single frame with the *y*-axis of each video, where 0 deg was negative *y* (Fig. 1B). Body angle was calculated at midstance (midstance θ_B_, deg) and rotation of the body (RoB, deg) was calculated by finding the difference between midstance θ_B_ of consecutive strides. The heading angle (θ_H_, deg) of each stride within a sequence was calculated by using the line that connects consecutive nose points relative to negative *y* (Fig. 1A).

Change of heading (CoH, deg) was calculated as the difference between the heading angles of consecutive strides, adjusting for the direction of the turn so that the difference was always positive (clockwise, counterclockwise). CoH was converted from degrees to radians and divided by the time between consecutive midstances to calculate angular velocity (ω, rad s^−1^). Turn radius (*r*, m) was calculated as *r*=*v*/ω, and centripetal acceleration (*a*_c_, m s^−2^) was calculated as *a*_c_=*v*ω, where *v* is stride velocity.

The coefficient of friction can be estimated by assuming that a successful turn of specific speed and radius requires a minimum friction (Alexander, 1982). Following these methods, a sequence in 0.05 increments from 0.01 to 4.96 was used as expected friction limits on the animals. Predicted angular velocity at each friction limit (ω_exp_, rad s^−1^) was compared with the recorded angular velocity (ω_actual_, rad s^−1^) taking the 95% upper limit of turns where ω_actual_ exceeds ω_exp_.

### Arrival and departure of turns

To compare similar strides within a turn, strides were given alphanumeric identifiers. The most severe CoH within a stride was considered the Apex of the turn. The last stride before entering the Apex was given Arrival (A)1 and the first stride exiting the Apex was given Departing (D)1. Strides within a turn increased distally (e.g. A2, A1, Apex, D1, D2).

### Performance density maps

We used the relative overlap between predator and prey performance landscapes to derive an estimated escape probability. This metric represents the likelihood of escape predicted from comparative locomotor performance rather than an empirically observed probability based on actual pursuit outcomes. For simplicity, we refer to this metric throughout as estimated escape probability. A performance density map containing all dogs was created with velocity (*v*, m s^−1^) on the *x*-axis and angular velocity (ω, rad s^−1^) on the *y*-axis. A kernel density contour plot was computed over the data using ‘kde’ from the ks package and ‘with’ from base R (https://CRAN.R-project.org/package=ks). This step was repeated for densities in a sequence of 10 to 95 in increments of 5. The contour data was extracted and converted to a shape file using the functions ‘Polygon’, ‘Polygons’ and ‘SpatialPolygons’ from the package sp (https://CRAN.R-project.org/package=sp). For the dog contour plot at 10%, a performance density map of the same variables (*x*=velocity, *y*=angular velocity) was created for each quoll subject and a kernel density contour of 10% was computed over the data. The area of overlap of each quoll subject's contour at 10% with the overall dog contour at 10% was calculated using ‘intersect’ from the package raster and the overlap of all individuals was averaged (https://CRAN.R-project.org/package=raster). This step was repeated for quoll contour plots from 10% to 95% using the 10–95% dog contour plots. This process was repeated for bandicoots using velocity and angular velocity and then again for both prey species using tangential acceleration and centripetal acceleration to create heatmaps.

### Statistical analyses

All data were analysed and processed in R version 4.0.5 (2021-03-31) (https://www.r-project.org/). Quantile regressions were used to analyse data that possessed a limitation boundary; such analyses used the function ‘rq’ from the package quantreg (v.5.94) (https://CRAN.R-project.org/package=quantreg). Quantile regressions were calculated using the upper or lower 5% quantile that represented the boundary limitations. The quantile regression analysis was used for angular velocity and turn radius. Linear mixed-effects models were used to analyse all other data when using the function ‘lme’ from the package nlme (https://CRAN.R-project.org/package=nlme).

For the performance landscape heatmaps, the effort differential (predator effort−prey effort) was calculated for each box that fell along the >55% estimated escape probability line. The estimated escape probability at >55% indicates the prey species has a greater chance of escaping than of being caught. Effort differentials and density plots were compared using Welch's *t*-tests.

Density curves of velocity, angular velocity, turn radius, CoH, RoB, θ_B_, *a*_t_ and *a*_c_ were calculated for each species and the range and height were used to compare the variability of strides as an approximation for predictability. Each range was converted to a proportion of the mean to standardise between different units. The ranges and heights of density curves were compared using Welch's *t*-tests.

Linear mixed-effects models were used to compare equivalent strides within a turning sequence, with centripetal acceleration, tangential acceleration, velocity, angular velocity, stride length and stride frequency treated as separate dependent variables. Species and stride position were included as fixed effects, and subject was included as a random effect. Pairwise comparisons of estimated marginal means were performed using the package emmeans (Table S1) (https://CRAN.R-project.org/package=emmeans).

## RESULTS

### Comparative speed and turning metrics

The cornering manoeuvres of three species were analysed and compared to help understand the contribution of locomotor performance to escape probability. Turning performance reflects the combined capacity to generate tangential and centripetal acceleration; consequently, speed, turn radius, angular velocity and body reorientation all contribute to overall performance. Overall, we analysed 387 strides from 29 bandicoots, 467 strides from 17 quolls, and 869 strides from 20 dogs. Dogs were much faster than both prey species, with a mean (±s.d.) speed of 3.55±1.27 m s^−1^ and observed maximum of 8.08 m s^−1^. Quolls showed a mean speed of 2.56±0.74 m s^−1^ with a maximum speed of 4.33 m s^−1^, and bandicoots’ mean speed was 2.38±0.76 m s^−1^ with a maximum speed of 4.84 m s^−1^ (Fig. 2). Dogs increased stride length (slope=−0.33, *R*^2^=0.81; Fig. 2H) to increase speed but did not adjust stride frequency (slope=−0.02, *R*^2^=0; Fig. 2I). Quolls favoured modulation of stride frequency (slope=0.63, *R*^2^=0.10; Fig. 2I) to increase speed and showed the least amount of adjustment to stride length (slope=0.12, *R*^2^=0.54; Fig. 2H), while bandicoots modulated both frequency and length at similar rates to increase speed (frequency: slope=0.23, *R*^2^=0.04; length: slope=0.21, *R*^2^=0.64; Fig. 2H,I). The interaction between species and both stride length and stride frequency was significant between dogs and prey species (bandicoot frequency: *t*_1717_=3.94, *P*<0.001; quoll frequency: *t*_1717_=10.94, *P*<0.001; bandicoot length: *t*_1717_=−10.4, *P*<0.001; quoll length: *t*_1717_=−18.8, *P*<0.001).

**Fig. 2.**
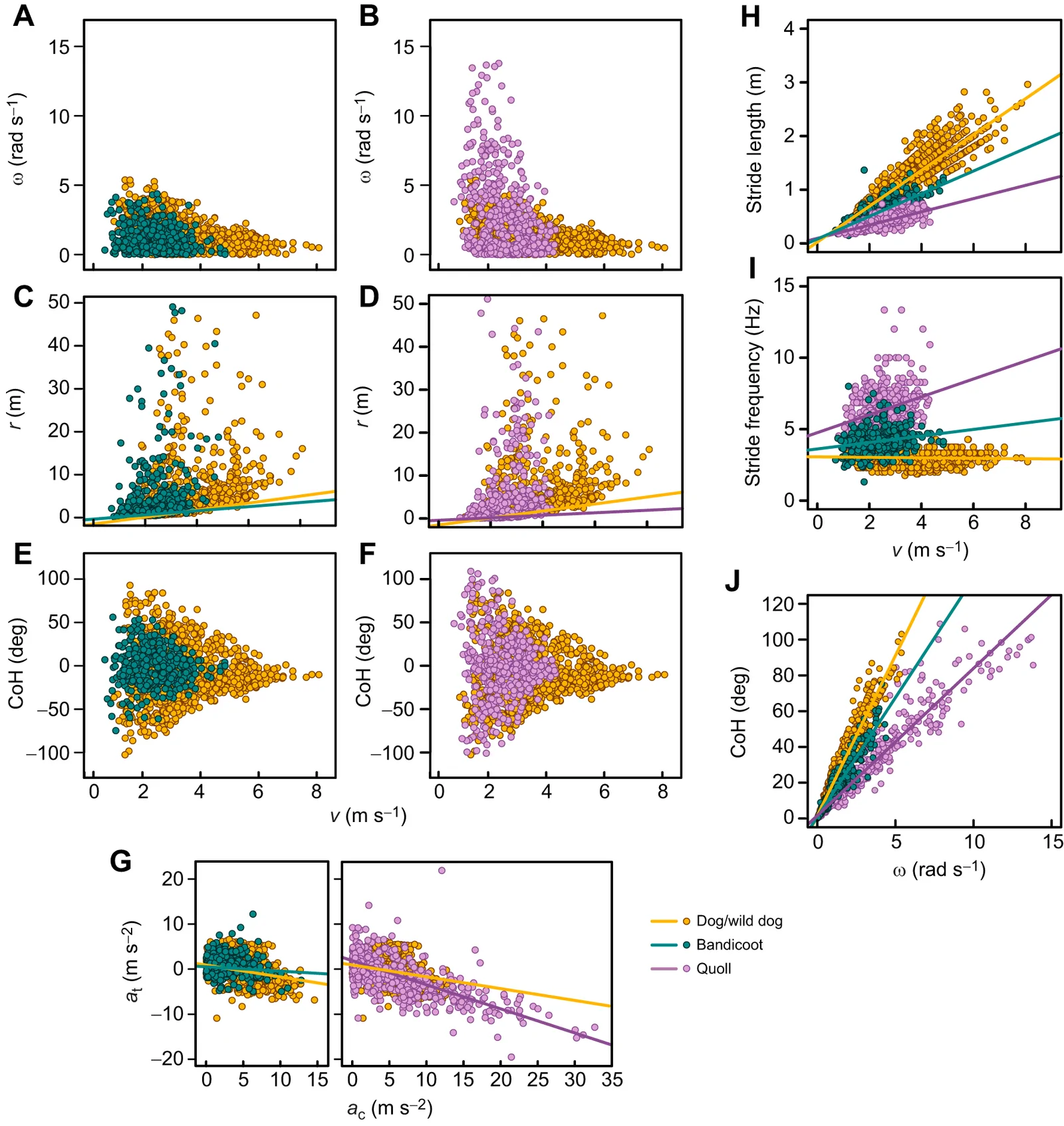
**The turning variables of individual strides in northern brown bandicoots and northern quolls in comparison to dogs.** (A,B) Angular velocity (ω), (C,D) turn radius (*r*) and (E,F) change of heading (CoH) against velocity for bandicoots (left, teal) and northern quolls (right, purple) in comparison to dogs (yellow). (G) Tangential acceleration (*a*_t_) and centripetal acceleration (*a*_c_). (H) Stride length and (I) stride frequency against velocity. (J) CoH compared with angular velocity. Fifth-quantile regressions representing the lower performance boundary are shown in C and D. Regression lines for each species are shown in G–J. *n*=387 strides from 29 bandicoots (teal), *n*=467 strides from 17 quolls (purple) and *n*=869 strides from 20 dogs (yellow).

Quolls had the highest average angular velocity (quolls: 2.83±2.99 rad s^−1^; dogs: 1.43±1.07 rad s^−1^; bandicoots: 1.18±0.95 rad s^−1^; Fig. 2A,B). The slopes of the lower limit (5%) of turning radii were compared among species because of the limitations of turning (as speed increases, turning radius increases; Fig. 2C,D). Quolls showed the shallowest slope of all three species (slope=0.29; lower 0.20, upper 0.36) and were significantly different from bandicoots (slope=0.48; lower 0.41, upper 0.64) and dogs, which had the highest slope (slope=0.81; lower 0.62, upper, 0.91). Quolls and dogs were able to change the heading of their turns up to a maximum of 109 deg and 103 deg within a single stride, respectively (Fig. 2F), while values for bandicoots were much lower (75 deg; Fig. 2E). Despite this, bandicoots (slope=13.35; Fig. 2J) adjusted their CoH at a faster rate than dogs (slope=17.88), and quolls adjusted their CoH at a faster rate than both (slope=8.23; Fig. 2J). Quolls showed significantly greater ability to produce centripetal acceleration than dogs (Fig. 2G). Quolls’ centripetal acceleration (slope=−0.53; *R*^2^=0.47; Fig. 2G) was negatively correlated to tangential acceleration and was significantly steeper than that of both dogs and bandicoots (dogs slope=−0.30; bandicoots slope=−0.13; *t*_1717_=−6.20).

### Coefficient of friction

Quolls showed a significantly higher coefficient of friction (1.96±0.57) than dogs (0.88±0.22; *t*_20.24_=−7.29, *P*<0.001) and bandicoots (0.69±0.22; *t*_18.95_=−8.75, *P*<0.001) (Fig. 3). Dogs and bandicoots were also significantly different (*t*_39.15_=2.90, *P*=0.007). The increased coefficient of friction of quolls could explain their increased turning ability, while the poor coefficient of friction for bandicoots could explain their performance regarding speed and manoeuvrability.

**Fig. 3.**
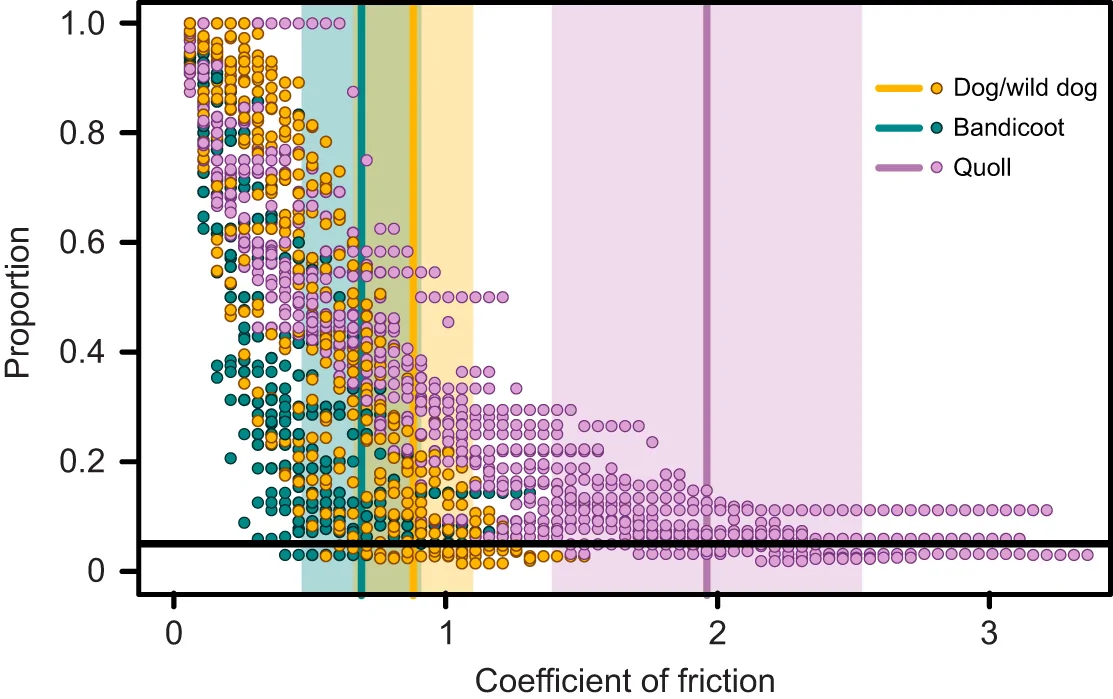
**The coefficient of friction for individual strides predicted for northern brown bandicoots, dogs and northern quolls.** Vertical lines and shading represent the mean and s.d. for each species. *n*=387 strides from 29 bandicoots (teal), *n*=467 strides from 17 quolls (purple) and *n*=869 strides from 20 dogs (yellow).

### Contour overlap landscape analysis

The performance capabilities of dogs, quolls and bandicoots were further explored by comparing the overlap between density contour plots of all dogs and averaged individual prey subjects (Fig. 4). This analysis showed the comparative effort and estimated escape probability between prey and predator. Quolls showed a high estimated escape probability when comparing the performance landscape of tangential and centripetal acceleration; however, this was reduced somewhat when comparing angular velocity and velocity (Fig. 4B,C). Bandicoots showed minimal estimated escape probability in both velocity and acceleration performance landscapes (Fig. 4D,E). For quolls, in both performance landscapes, the 55% estimated escape probability exceeded the line of equivalent effort (black), while in bandicoots, this was reduced. The effort differential (predator effort−prey effort) was calculated to understand how much effort is required to achieve a 55% estimated escape probability. For acceleration landscapes, quolls were able to achieve a 55% estimated escape probability by using 14±9.5% less effort than dogs, while bandicoots used 36±11.2% more effort to achieve the same estimated escape probability (*t*_26.13_=−14.45, *P*<0.001; Fig. 4B,D). For velocity landscapes, quolls required 3.2±8.0% less effort to achieve a 55% escape probability and bandicoots required 25.8±10.8% more effort (*t*_34.21_=−9.99, *P*<0.001; Fig. 4C,E). Quolls showed decreased effort differentials in acceleration landscapes, while bandicoots showed decreased effort differentials in velocity performance landscapes (*t*_45_=−4.35, *P*<0.001; Fig. 4B,C; *t*_29.6_=2.80, *P*=0.008; Fig. 4D,E). Using these estimations, quolls seem to escape predation using much less effort than would be required by dogs, while bandicoots need to use close to maximum effort to escape dogs that do not exhibit full running capacity.

**Fig. 4.**
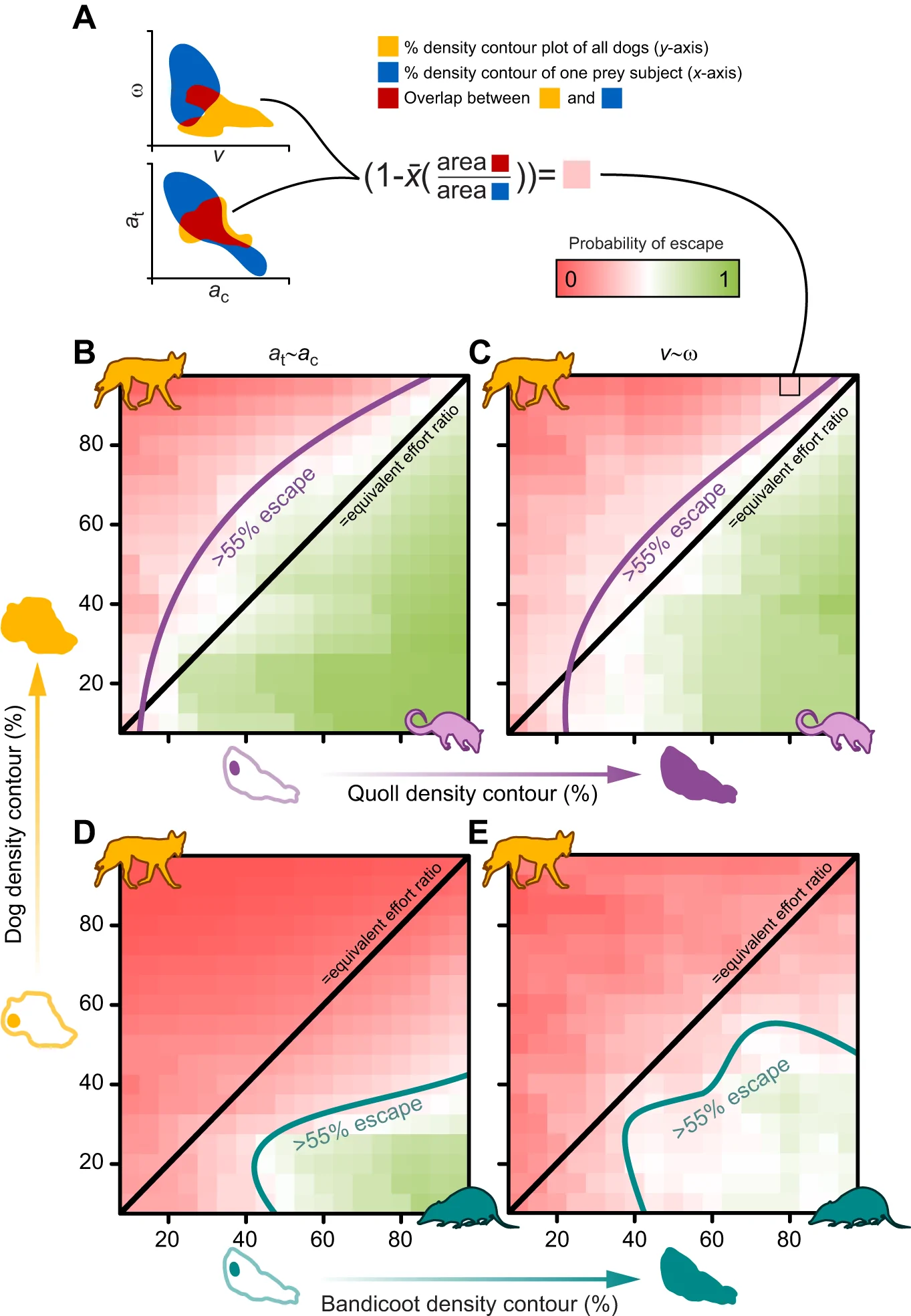
**Heatmaps displaying the results of a contour overlap landscape analysis comparing dog performance landscapes with those of northern quolls and northern brown bandicoots.** (A) Method and equation used to create each cell within the heatmaps and the heatmap key (green, escape; red, capture). (B,D) Dog density contour against those for quoll (B) and bandicoot (D), created from performance landscapes using tangential and centripetal acceleration (m s^−2^). (C,E) Dog density contour against those for quoll (C) and bandicoot (E), created from performance landscapes using velocity (m s^−1^) and angular velocity (rad s^−1^). The *x*- and *y*-axes represent the percentage density contour for predator and prey to reproduce each square within the heatmaps. *n*=387 strides from 29 bandicoots (teal), *n*=467 strides from 17 quolls (purple) and *n*=869 strides from 20 dogs (yellow). *a*_t_, tangential acceleration; *a*_c_, centripetal acceleration; *v*, velocity; ω, angular velocity.

### Densities for predictability

Density plots were analysed to show the variability of choices during turning (Fig. 5). Bandicoots showed a smaller proportional range (0.70±0.28) and a higher maximum (0.22±0.20) than both quolls (range 1.18±0.46, maximum 0.14±0.15) and dogs (range 1.12±0.51, maximum 0.16±0.15). However, only the proportional range between quolls and bandicoots showed a significant difference (*t*_11.5_=−2.467, *P*=0.03). Velocity and CoH were the only two performance metrics where bandicoots showed lower peaks than quolls; however, quolls either showed a similar or broader range in both plots (Fig. 5A,D). Dogs showed a broader range in velocity and turn radius than the other species. The limited range and increased maximum of bandicoot density plots suggests that their escape trajectories would be more predictable than those of quolls.

**Fig. 5.**
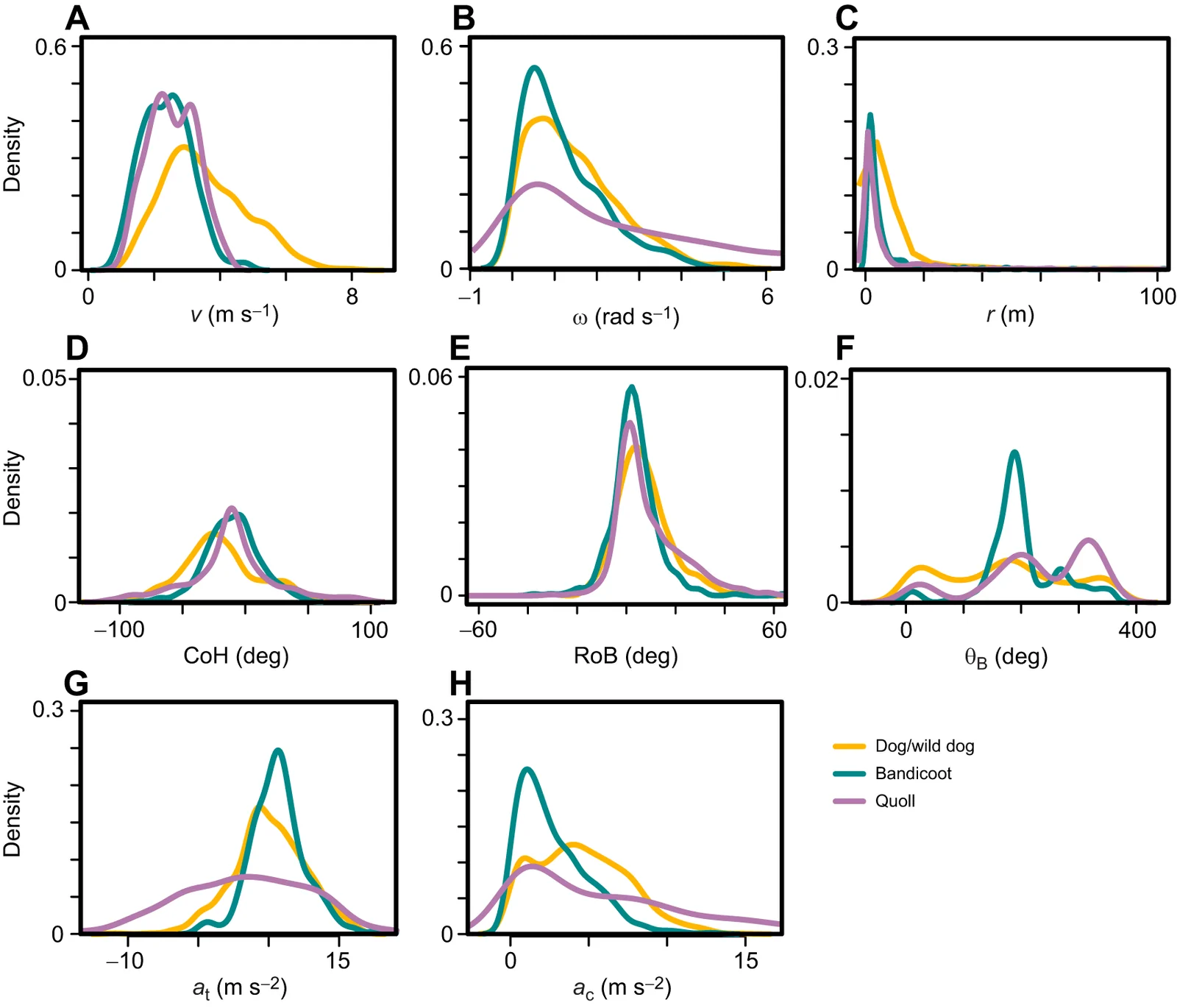
**Density plots of performance metrics used for turning analysis of dogs, northern brown bandicoots and northern quolls.** (A) Velocity (*v*), (B) angular velocity (ω), (C) turn radius (*r*), (D) change of heading (CoH), (E) rotation of the body (RoB), (F) body angle (θ_B_), (G) tangential acceleration (*a*_t_) and (H) centripetal acceleration (*a*_c_). *n*=387 strides from 29 bandicoots (teal), *n*=467 strides from 17 quolls (purple) and *n*=869 strides from 20 dogs (yellow).

### Equivalent stride analysis

Equivalent strides within a turn were compared (Fig. 6). There was a significant effect of species on all variables, with the greatest effect on stride frequency (*a*_t_: *f*_2,61_=86.21, *P*<0.001; *a*_c_: *f*_2,61_=46.85, *P*<0.001; *v*: *f*_2,61_=22.18, *P*<0.001; ω: *f*_2,61_=95.52, *P*<0.001; stride length: *f*_2,88_=80.80, *P*<0.001; stride frequency: *f*_2,111_=154.05, *P*<0.001). Stride position had a significant effect on all six variables, with the largest *F*-statistic observed for angular velocity (*a*_t_: *f*_6,1637_=117.52, *P*<0.001; *a*_c_: *f*_6,1637_=171.15, *P*<0.001; *v*: *f*_6,1637_=112.39, *P*<0.001; ω: *f*_6,1637_=191.09, *P*<0.001; stride length: *f*_6,1634_=31.68, *P*<0.001; stride frequency: *f*_6,1634_=16.48, *P*<0.001). Quolls displayed the highest centripetal acceleration at the apex of the turn, while bandicoots showed a significantly lower centripetal acceleration at the apex (Fig. 6A). Quolls showed more negative tangential acceleration than dogs when entering the apex but accelerated at similar rates when departing. Bandicoots showed greater tangential acceleration through the apex of the turn, which was significant compared with both dogs and quolls (Fig. 6B). Quolls had similar speeds to dogs when approaching the apex but showed significantly slower speeds departing the apex (Fig. 6C). Bandicoots showed significantly lower angular velocity in strides A1, Apex and D1 in comparison to dogs, and quolls showed a significantly higher angular velocity at the apex (Fig. 6D). During a turning sequence, bandicoots were slower when approaching the apex (A3–A1) and did not use as much negative tangential acceleration. While their velocity was comparable to that of both dogs and quolls during strides at the apex and D1, they did not seem to increase tangential acceleration or speed when departing the apex (D1–D3). Dogs showed increased stride length and decreased stride frequency compared with both prey species throughout the turns (Fig. 6E,F; also see Fig. 2H,I).

**Fig. 6.**
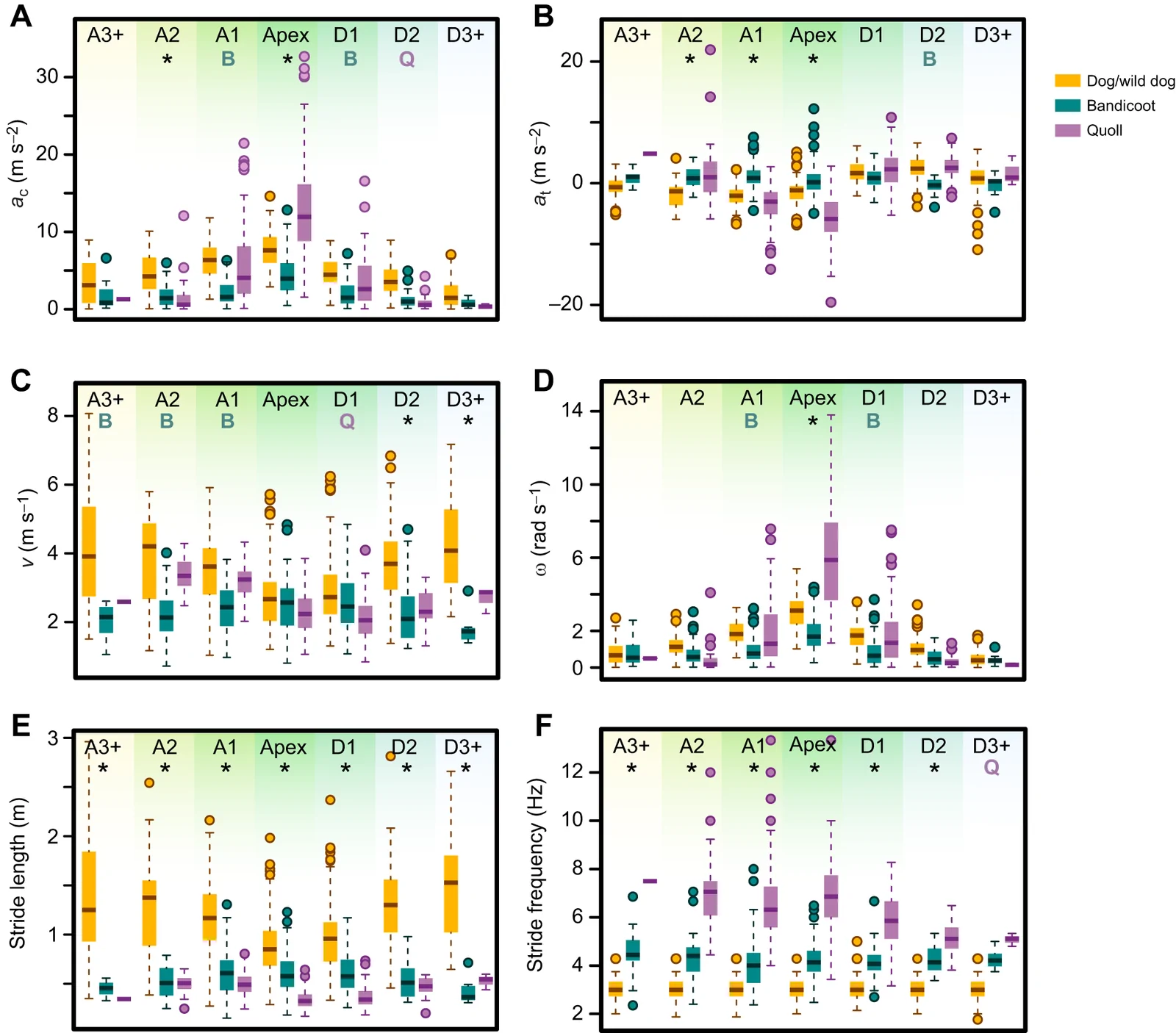
**Comparative strides within a turn of dogs, northern brown bandicoots and northern quolls. (A) Centripetal acceleration (*a*_c_), (B) tangential acceleration (*a*_t_), (C) velocity (*v*), (D) angular velocity (ω), (E) stride length (m) and (F) stride frequency.** Data are for the stride with the largest CoH (apex) and strides arriving at the apex (A3+, A2, A1) and departing from the apex (D1, D2, D3+). Box plots show median, upper and lower quartiles and 1.5× the interquartile range. Black asterisks show that both species differ significantly from dogs, teal B indicates that only bandicoots differ, and purple Q indicates that only quolls differ. *n*=387 strides from 29 bandicoots (teal), *n*=467 strides from 17 quolls (purple) and *n*=869 strides from 20 dogs (yellow). Comparative strides were analysed using linear mixed-effects models.

## DISCUSSION

### The natural experiment

Our original hypothesis was that the bandicoots should possess a greater overall locomotor performance of the prey species; however, quolls showed greater capacity for locomotion. Based on locomotor performance, bandicoots were slower in comparison to quolls. The contour overlap analysis showed that bandicoots had a low estimated escape probability when pursued by dogs, while quolls had a higher estimated escape probability, mainly through their ability to adjust their change of heading (CoH) at faster rates than dogs. The locomotor performance of quolls, in comparison to that of dogs, supports findings for Groote Eylandt populations, where quolls show high naive occupancy estimates, and turning ability is proportional to recruitment into successive breeding seasons (Rew-Duffy et al., 2020; Heiniger, Davies and Gillespie, 2020a). Bandicoots on Groote Eylandt also have high naive occupancy estimates despite their poorer estimated escape probabilities relative to canids present on the island (Heiniger, Davies and Gillespie, 2020a). From the same study, both species showed reduced observations on mainland Australia, but quoll estimates were reduced by approximately 98.47% as opposed to 52.9% in bandicoots, which does not reflect the locomotor performance of these two species. This reveals much about Groote Eylandt, a remote island which holds pristine habitat free from foxes (*Vulpes vulpes*), cane toads (*Rhinella marina*), habitat degradation due to invasive herbivores, and suppressed presence of cats, all things that cause declines in these fauna (Oakwood, 1997; Heiniger et al., 2020b). How much then does locomotion and escaping predation contribute to these species at a population level?

### Does locomotion contribute to northern quoll survival rate?

While quolls have a greater turning ability, their low top speed in comparison to dogs would leave them vulnerable in situations during unobstructed straight running. Wilson et al. (2015) showed that mistimed turns by prey would result in the predator ‘cutting corners’, reducing the effect of size limitations for turning. Without cutting corners, the predator must match the angular velocity of the prey or risk overshooting the turn, increasing the distance between prey and predator (Wilson et al., 2015). On the mainland, quolls are in greater densities in highly complex environments (rocky outcrops) and are less commonly found in open woodlands or where habitat complexity has been reduced (Oakwood, 1997; Oakwood et al., 2016). Some predators, such as wolf spiders and cheetahs, experience reduced hunting success when hunting in complex habitats (Mills et al., 2004; Morice et al., 2013). The benefit of habitat complexity is not completely understood, with suggested theories of visual or physical obstruction, refuge abundance or resource abundance (Bonenfant and Kramer, 1996; Laundré, Hernández and Ripple, 2010; Morice et al., 2013). Despite this, prey species such as quokkas (*Setonix brachyurus*) are found more commonly in complex habitats and are suggested to use these habitat types to limit predation from foxes (Hayward et al., 2005). For quolls, greater turning performance would have an increased effect in complex habitats where predators cannot cut corners and straight running is limited (Wilson et al., 2015; Rew-Duffy et al., 2020; Wheatley et al., 2020). This could explain greater densities of quolls reported in complex rocky outcrops, which would favour their increased manoeuvrability.

In comparison to quolls' preference for rocky habitat, bandicoots prefer dense ground vegetation in the shade of woodlands for both foraging and protection from predators; however, they are very adaptable and can inhabit a broad range of environments (Kemper et al., 1989; FitzGibbon, Wilson and Goldizen, 2011). On Groote Eylandt, quolls will occupy similar habitats with dense ground vegetation (Heiniger et al., 2020b). On mainland Australia, the destruction of dense vegetation through fires and invasive herbivores has caused declines in quolls, while bandicoots show more versatility (Oakwood, 1997; Kemper et al., 1989; FitzGibbon, Wilson and Goldizen, 2011).

### Does locomotion contribute to northern brown bandicoot survival rate?

Bandicoots performed poorly in comparison to quolls and dogs in terms of their turning ability, which could not be related to choosing submaximal speeds. Prey will often choose to run at submaximal speeds for the purpose of reducing mistakes and decreasing predictability (Wynn et al., 2015; Amir Abdul Nasir et al., 2017; Wilson et al., 2013). The average speeds recorded here for bandicoots are similar to average speeds recorded in other studies on bandicoot locomotion, and thus it is unlikely that they are limiting speed (Tay et al., 2022). Increasing the variability of the escape trajectory has also been strongly associated with survival rate of prey within pursuit interactions (Moore et al., 2017). However, when comparing the variability of turning parameters among species, bandicoots were the most predictable and are unlikely to be increasing variability in escape trajectories (Fig. 5). Bandicoots’ inability to outperform dogs, in combination with their predictable trajectory, makes them common targets for dingoes/wild dogs. This is reflected in the increased appearance of bandicoot-like species in dog diets when compared with dasyurids (including quolls); however, bandicoots still persist in large populations on mainland Australia (Gaschk and Clemente, 2022).

Bandicoots' ability to persist in broad distributions, despite the pressure of canid predation and their poor locomotor performance, could be related to many aspects of their ecology. Their reproductive strategy, which comprises 4–5 joeys per litter, a relatively short gestation/weaning period (∼72 days) and an annual fecundity of 4.7 litters per year, would allow populations to remain stable (Gemmell, Veitch and Nelson, 1999; FitzGibbon, 2015; Tay et al., 2022).

Bandicoots' survival could also be due to adaptable foraging behaviour to limit detection from predators or reduce distance to refuges (Lima and Dill, 1990). The landscape of fear is a concept that suggests animals will actively avoid foraging areas that are too far from known refuges or lack adequate complexity (Laundré, Hernández and Ripple, 2010). Bandicoots change their foraging behaviour in the presence of predator scents, increasing vigilance when foraging close to, or outright avoiding, the scented location (Bytheway and Banks, 2019). Bandicoots have also been shown to change their peak temporal activity period from bimodal to unimodal in response to rainfall variation, correlating with a change in diet (Diete et al., 2017). Thus, versatile foraging and reliable reproduction are likely to support population persistence through canid predation, while speed and manoeuvrability contribute less to individual survival.

### Dingoes and domestic dogs

Australian fauna experience predation from three forms of canids in Australia: domestic/wild dogs, dingoes and foxes. Dogs are a good approximation for the predation pressure from dingoes but also contribute to this pressure directly. Many of the differences between the skeletal morphology of dingoes and free-roaming feral dogs have been identified in the cranium; however, these differences are within the morphological range found in domestic dog breeds (Smith et al., 2019; Jackson et al., 2021; Koungoulos et al., 2024). Genomic comparisons of dingoes and domestic dogs show that the two are distinguishable genetically (Smith et al., 2019; Cairns et al., 2023; Souilmi et al., 2024). However, despite this distinguishable difference between dingoes and free-roaming feral dogs, they share a similar hunting ecology within comparable combinations of ecosystems and conditions; they are opportunistic hunters with commensal behaviours under certain circumstances (Jackson et al., 2021; Gaschk, 2023). With dingoes showing a similar hunting ecology, conditional sociality, indistinguishable morphology and historical interbreeding with Australian dog breeds, the range of domestic dogs used in this study likely encompasses the performance capability of dingoes (Smith et al., 2019).

### Friction, mass and substrate

Northern quolls showed the greatest turning capacity of the three species, which seemed to correlate with an increased coefficient of friction. Friction allows for a greater capacity to apply force to the substrate without slipping and is vital during turning because the force applied can be perpendicular to initial direction (Alexander, 1982; Haagensen et al., 2022). Quoll manoeuvrability is facilitated by their small size, crouched posture and foot pad morphology, allowing them to deflect the centre of mass from its original heading (momentum) to the new direction (Alexander, 1982; Wynn et al., 2015; Wheatley et al., 2018). Further, their ability to modulate stride frequency increases the potential number of centre of mass deflections within a turning sequence, resulting in higher angular velocities and more severe CoHs.

Dogs only modulate stride length when changing speed; when gaining speed, this limits the opportunity for deflection of the centre of mass during a turn and increases the distance between these deflections. Dogs also have to contend with a much higher centre of mass and greater momentum through the turns because of their larger size and more upright posture (Haagensen et al., 2022). Bandicoots, in contrast, have a crouched posture and are much smaller, lowering their centre of mass, and as such should have an advantage in turning ability. Despite their crouched posture and smaller body size, bandicoots had the lowest coefficient of friction (Fig. 3). However, the coefficient of friction of both dogs and bandicoots was far lower than that of quolls. This could be explained by the type of substrate used in these experiments.

Quolls performed their trials on a thin rubber matting, while bandicoots and dogs completed their trials on a grassy-soil substrate, with some bandicoots running on a loamy soil. A study looking at the task of pulling sleds across substrates found that the coefficient of friction for a grass pitch was 0.45±0.1 while on a rubber athletics track it was 0.58±0.1 (Linthorne and Cooper, 2013). When comparing performance across the two substrates, the rubber surface generated 29% more friction than the grass surface. Applying this correction to the coefficient of friction recorded for quolls on rubber (1.96/1.29) yields an estimated value of 1.52 for turns performed on grass. This remains substantially higher than the values estimated for both dogs and bandicoots. Therefore, although the rubber matting provides some mechanical advantage, it does not materially alter the overall result.

### Conclusion

We explored the effect of locomotor performance on the escape probability of native Australian species from canid predation. Dogs showed higher top speeds, quolls showed better manoeuvrability and unpredictability, while bandicoots were wholly outperformed. For bandicoots, locomotor performance does not seem to contribute to population survival, which is likely related to other aspects of their ecology. Locomotor performance for quolls, particularly their manoeuvrability, supports their use of complex habitats (e.g. rocky outcrops) to escape pursuers. Future research is needed to determine whether performance is a reliable predictor of population survival in species that have similarly receded to refuge habitats (e.g. quokkas). This study highlights that locomotor performance alone may not be able to accurately predict the abundance or distribution of populations, despite being a key determinant in the outcomes of predator–prey interactions.

## Supplementary Material

10.1242/jexbio.252204_sup1 Supplementary information
