# Supplementary material for: Pursuit dynamics among Australian marsupials and dogs: comparative locomotor performance and escape probability
Source: J Exp Biol. 2026 Oct 5;229(19):jeb252204. doi: 10.1242/jeb.252204 (PMC13723406; doi:10.1242/jeb.252204)
Supplement: Supplementary file 1 [file jexbio-229-252204-s1.pdf]

**Table S1. The significant results from the Tukey post hoc test comparing turning variables with similar strides.** D- Dogs, B- Bandicoots, Q – Quoll. Data corresponds to Figure 6.

| Variable | Comparison | estimate | SE | df | t.ratio | p.value |
|---|---|---|---|---|---|---|
| Centripetal Acceleration ($m.s^{-2}$) | A2 - D:B | -2.98 | 0.63 | 485.89 | -4.70 | < 0.001 |
| Centripetal Acceleration ($m.s^{-2}$) | A2 - D:Q | 3.04 | 0.68 | 412.48 | 4.51 | 0.002 |
| Centripetal Acceleration ($m.s^{-2}$) | A1 - D:B | -3.88 | 0.48 | 190.39 | -8.09 | < 0.001 |
| Centripetal Acceleration ($m.s^{-2}$) | Apex - D:B | -3.15 | 0.45 | 152.15 | -6.97 | < 0.001 |
| Centripetal Acceleration ($m.s^{-2}$) | Apex - D:Q | -5.55 | 0.48 | 119.66 | -11.61 | < 0.001 |
| Centripetal Acceleration ($m.s^{-2}$) | D1 - D:B | -2.31 | 0.50 | 217.13 | -4.64 | 0.001 |
| Centripetal Acceleration ($m.s^{-2}$) | D2 - D:Q | 2.70 | 0.58 | 247.39 | 4.63 | 0.001 |
| Tangential Acceleration ($m.s^{-2}$) | A2 - D:B | 2.92 | 0.50 | 1503.37 | 5.89 | < 0.001 |
| Tangential Acceleration ($m.s^{-2}$) | A2 - D:Q | -3.76 | 0.52 | 1327.23 | -7.28 | < 0.001 |
| Tangential Acceleration ($m.s^{-2}$) | A1 - D:B | 3.19 | 0.33 | 1086.15 | 9.64 | < 0.001 |
| Tangential Acceleration ($m.s^{-2}$) | A1 - D:Q | 1.38 | 0.31 | 699.21 | 4.39 | 0.002 |
| Tangential Acceleration ($m.s^{-2}$) | Apex - D:B | 1.88 | 0.30 | 865.00 | 6.32 | < 0.001 |
| Tangential Acceleration ($m.s^{-2}$) | Apex - D:Q | 4.35 | 0.29 | 587.44 | 14.82 | < 0.001 |
| Tangential Acceleration ($m.s^{-2}$) | D2 - D:B | -3.05 | 0.56 | 1496.69 | -5.45 | < 0.001 |
| Velocity ($m.s^{-1}$) | A3 - D:B | -2.30 | 0.25 | 237.02 | -9.16 | < 0.001 |
| Velocity ($m.s^{-1}$) | A2 - D:B | -1.64 | 0.22 | 133.17 | -7.64 | < 0.001 |
| Velocity ($m.s^{-1}$) | A1 - D:B | -1.01 | 0.19 | 84.20 | -5.29 | < 0.001 |
| Velocity ($m.s^{-1}$) | D1 - D:Q | 0.79 | 0.21 | 74.48 | 3.78 | 0.040 |
| Velocity ($m.s^{-1}$) | D2 - D:B | -1.16 | 0.23 | 163.92 | -5.11 | < 0.001 |
| Velocity ($m.s^{-1}$) | D2 - D:Q | 1.26 | 0.22 | 94.84 | 5.68 | < 0.001 |
| Velocity ($m.s^{-1}$) | D3 - D:B | -1.94 | 0.31 | 479.24 | -6.32 | < 0.001 |
| Velocity ($m.s^{-1}$) | D3 - D:Q | 1.79 | 0.42 | 818.52 | 4.32 | 0.003 |
| Angular Velocity ($rad.s^{-1}$) | A1 - D:B | -0.90 | 0.18 | 357.50 | -4.92 | < 0.001 |

| | | | | | | |
|---|---|---|---|---|---|---|
| Angular Velocity (rad.s-1) | Apex - D:B | -1.16 | 0.17 | 269.60 | -6.85 | < 0.001 |
| Angular Velocity (rad.s-1) | Apex - D:Q | -3.17 | 0.17 | 190.34 | -18.29 | < 0.001 |
| Angular Velocity (rad.s-1) | D1 - D:B | -0.81 | 0.19 | 408.28 | -4.23 | 0.005 |
| Stride Length (m) | A3 - D:B | 1.02 | 0.08 | 209.68 | 12.03 | < 0.001 |
| Stride Length (m) | A3 - D:Q | 1.19 | 0.22 | 1500.02 | 5.48 | < 0.001 |
| Stride Length (m) | A2 - D:B | 0.78 | 0.07 | 122.30 | 10.71 | < 0.001 |
| Stride Length (m) | A2 - D:Q | 0.85 | 0.08 | 111.88 | 10.52 | < 0.001 |
| Stride Length (m) | A1 - D:B | 0.58 | 0.07 | 80.91 | 8.77 | < 0.001 |
| Stride Length (m) | A1 - D:Q | 0.71 | 0.07 | 73.45 | 9.79 | < 0.001 |
| Stride Length (m) | Apex - D:B | 0.30 | 0.06 | 75.44 | 4.69 | 0.002 |
| Stride Length (m) | Apex - D:Q | 0.58 | 0.07 | 70.81 | 8.07 | < 0.001 |
| Stride Length (m) | D1 - D:B | 0.35 | 0.07 | 85.09 | 5.27 | < 0.001 |
| Stride Length (m) | D1 - D:Q | 0.64 | 0.07 | 72.62 | 8.87 | < 0.001 |
| Stride Length (m) | D2 - D:B | 0.72 | 0.08 | 148.09 | 9.32 | < 0.001 |
| Stride Length (m) | D2 - D:Q | 0.86 | 0.08 | 90.00 | 11.25 | < 0.001 |
| Stride Length (m) | D3 - D:B | 0.94 | 0.10 | 413.78 | 9.23 | < 0.001 |
| Stride Length (m) | D3 - D:Q | 1.05 | 0.14 | 713.75 | 7.68 | < 0.001 |
| Stride Frequency (Hz) | A3 - D:B | -1.41 | 0.26 | 539.61 | -5.49 | < 0.001 |
| Stride Frequency (Hz) | A3 - D:Q | -4.59 | 0.78 | 1685.06 | -5.86 | < 0.001 |
| Stride Frequency (Hz) | A2 - D:B | -1.27 | 0.20 | 269.34 | -6.26 | < 0.001 |
| Stride Frequency (Hz) | A2 - D:Q | -4.06 | 0.22 | 232.13 | -18.56 | < 0.001 |
| Stride Frequency (Hz) | A1 - D:B | -1.14 | 0.16 | 123.79 | -6.90 | < 0.001 |
| Stride Frequency (Hz) | A1 - D:Q | -3.72 | 0.17 | 98.77 | -21.30 | < 0.001 |
| Stride Frequency (Hz) | Apex - D:B | -1.17 | 0.16 | 105.76 | -7.41 | < 0.001 |
| Stride Frequency (Hz) | Apex - D:Q | -3.87 | 0.17 | 90.61 | -22.60 | < 0.001 |
| Stride Frequency (Hz) | D1 - D:B | -1.14 | 0.17 | 137.22 | -6.74 | < 0.001 |
| Stride Frequency (Hz) | D1 - D:Q | -2.97 | 0.17 | 95.73 | -17.09 | < 0.001 |
| Stride Frequency (Hz) | D2 - D:B | -1.28 | 0.22 | 358.81 | -5.77 | < 0.001 |
| Stride Frequency (Hz) | D2 - D:Q | -2.41 | 0.20 | 153.14 | -12.31 | < 0.001 |
| Stride Frequency (Hz) | D3 - D:Q | -2.42 | 0.47 | 1452.88 | -5.16 | < 0.001 |

Journal of Experimental Biology • Supplementary information

**Table S2.** A list of dog breeds used in this study from (Haagensen *et al.* 2022)

| Breed | Number |
|---|---|
| **Papillon** | 1 |
| **Portuguese Podengo Pequeno** | 1 |
| **Cavalier King Charles** | 2 |
| **Border Collie** | 8 |
| **Tasmanian Smithfield** | 1 |
| **German Coolie** | 1 |
| **Australian Shepherd** | 1 |
| **Bearded Collie** | 1 |
| **Retriever** | 1 |
| **Belgium** Malinois | 1 |
| **German Shepherd** | 1 |
| **Mixed Breed** | 1 |
